# Assessing the Effectiveness and Cost-Benefit of Test-and-Vaccinate Policy for Supplementary Vaccination against Rubella with Limited Doses

**DOI:** 10.3390/ijerph15040572

**Published:** 2018-03-22

**Authors:** Masaya M. Saito, Keisuke Ejima, Ryo Kinoshita, Hiroshi Nishiura

**Affiliations:** 1 The Institute of Statistical Mathematics, 10-3 Midori-cho, Tachikawa, Tokyo 190-8562, Japan; saitohm@ism.ac.jp; 2 Core Research for Evolutional Science and Technology (CREST), Japan Science and Technology Agency, 4-1-8 Honcho, Kawaguchi, Saitama 332-0012, Japan; kinoshitaryo@gmail.com; 3 Institute of Industrial Science, The University of Tokyo, Tokyo 153-8505, Japan; kejima@iu.edu; 4 School of Public Health, Indiana University Bloomington, 1025 E 7th St #111, Bloomington, IN 47405, USA; 5 Graduate School of Medicine, Hokkaido University, Kita 15 Jo Nishi 7 Chome, Kita-ku, Sapporo, Hokkaido 060-9638, Japan

**Keywords:** rubella, vaccination, serology, cost-benefit, epidemic, mathematical model, Japan

## Abstract

Elevating herd immunity level against rubella is essential to prevent congenital rubella syndrome (CRS). Insufficient vaccination coverage left susceptible pockets among adults in Japan, and the outbreak of rubella from 2012 to 2013 resulted in 45 observed CRS cases. Given a limited stock of rubella-containing vaccine (RCV) available, the Japanese government recommended healthcare providers to prioritize vaccination to those confirmed with low level of immunity, or to those likely to transmit to pregnant women. Although a test-and-vaccinate policy could potentially help reduce the use of the limited stockpile of vaccines, by selectively elevating herd immunity, the cost of serological testing is generally high and comparable to the vaccine itself. Here, we aimed to examine whether random vaccination would be more cost-beneficial than the test-and-vaccinate strategy. A mathematical model was employed to evaluate the vaccination policy implemented in 2012–2013, quantifying the benefit-to-cost ratio to achieve herd immunity. The modelling exercise demonstrated that, while the test-and-vaccinate strategy can efficiently achieve herd immunity when stockpiles of RCV are limited, random vaccination would be a more cost-beneficial strategy. As long as the herd immunity acts as the goal of vaccination, our findings apply to future supplementary immunization strategy.

## 1. Introduction

Rubella, caused by infection with rubella virus, is generally a mild and self-limiting disease, but infection during pregnancy, especially during the first trimester, can result in complications in the fetus, known as the congenital rubella syndrome (CRS) [1]. The pitfall of mass vaccination strategies against rubella is that, when the population is not sufficiently immunized to prevent a major epidemic, vaccination allows a push forward of the age at infection. Thus, when the risk of rubella infection is elevated to women at childbearing age, vaccination and its insufficient herd immunity can controversially increase the risk of CRS in the fetus [2,3]. For a long time, sufficiently elevating herd immunity level has been recognized as an essential step to prevent rubella and CRS, and also, to medically and economically justify the implementation of mass vaccination [4,5].

In Japan, routine vaccination against rubella has been conducted since 1977 [6]. Until March 1995, the subject of vaccination was focused on women aged from 12 to 15 years. From April 1995, following the revision of Immunization Law in 1994, the policy was extended to cover additional subjects, regardless of gender, including infants 12 to 90 months old, shifting the policy from individual protection, to elevating herd immunity. Since April 2006, a combined measles and rubella (MR) vaccine started to be used, and the second dose has also been initiated. The subject age groups were then changed so that the vaccination can be completed before school age; 12 to 24 months for the first dose, and 48 to 72 months for the second dose. Due to the changes in vaccination policy, in Japan, immunity status varies by birth-year cohort and gender. Specifically, seroepidemiological surveillance has identified susceptible pockets that still remained among adult males who missed the opportunity to obtain vaccine-induced or naturally-acquired immunity, e.g., susceptible pockets among male birth cohorts 1989–1993 and 1974–1978 were identified, with seropositive proportions of 70.0% and 68.0%, respectively [7]. In majority of industrialized countries, there is no such gap cohort, and substantial herd immunity to prevent a major epidemic is considered to have been achieved, following mass vaccination among infants. Thus, a supplementary vaccination program targeting fully susceptible adult populations is deemed essential to achieve a sufficient level of herd immunity and avoid another major epidemic (i.e., the epidemic that does not probabilistically decline to extinction) in the future, or at least mitigate the epidemic burden.

While vaccination policy is judged by various aspects, including laboratory test performance and adverse events of vaccination, we focus on the supplementary vaccination policy which is implemented as a reaction to the re-emergence of rubella. If the total amount of available vaccines is sufficient to cover the whole population (regardless of immunity status), or at least enough doses to achieve herd immunity, the best practice would be to randomly distribute as many vaccines as possible. However, in case the available maximum doses are limited, which actually happened in Japan, 2012–2013, the goal will be to efficiently allocate the vaccine by identifying an optimal strategy to minimize the burden. Here, we introduce the experience of rubella control in 2012–2013. In 2012, an outbreak of rubella was observed, but was thought to be over by the end of the year, due to the decreasing number of cases (2386 cases). However, the situation changed from the start of 2013 in Japan, as an increasing number of rubella cases were observed again, reaching to 14,344 confirmed rubella cases by the end of the year [8,9]. The epidemic provoked the increasing demand and awareness regarding vaccine uptake against rubella. In June, the Japanese Ministry of Health, Labour and Welfare (MHLW) estimated that vaccine stockpiles of rubella-containing vaccine (RCV) would run short by autumn [10]. Given this forecast, the MHLW requested medical institutions to prioritize vaccination to (1) those who have insufficient antibody titer (i.e., the so-called “test-and-vaccination” policy), and (2) those around women presently or planning to be pregnant. In July, the MHLW also requested prefectural governments to financially support immunizing these prioritized people [11].

Above, we had two different target populations for supplementary vaccination; however, let us focus on the first population which must undertake antibody testing in advance of vaccination, and requires additional cost to vaccinate due to negative laboratory assay. Even provided that more than sufficient amounts of test kits were available, this test-and-vaccinate policy might not be economically justified compared with random mass vaccination policy, in which the pre-immunization test is never performed, and randomly selected individuals undertake vaccination regardless of the history of vaccination or natural infection.

Using a mathematical model, the objective of the present study is to be the first to assess and compare the effectiveness (i.e., how much the cumulative incidence can be averted compared with the absence of supplementary vaccination) and cost-benefit (i.e., monetary expenditure vs gain from the implementation of vaccination) of the two different vaccination policies: (1) random mass vaccination (i.e., population-wide vaccination of subject people, regardless of immune status) and (2) vaccination following rubella antibody testing (or hereafter, we refer to it as selective vaccination or its policy as the “test-and-vaccine policy”). For comparative purposes, we also simulate the scenario where no supplementary vaccination takes place.

## 2. Materials and Methods

### 2.1. Rubella Epidemic Model

Here we use two different outcomes, i.e., the cumulative incidence of an epidemic and benefit-to-cost ratio, and compare them by different vaccination programs. In this section, we construct the rubella epidemic model, modifying the so-called “Susceptible-Infected-Removed” (SIR) model, which is commonly devised to explain the epidemic dynamics of directly transmitted infectious diseases [12], while we additionally account for vaccination program in the following. The detail of mathematical implementation of each supplementary vaccination policy is explained in the next section.

Given that vaccinated individuals are still at risk of infection, the susceptible population is composed of two groups, those vaccinated (partially susceptible, i.e., immunized but subject to infection upon exposure with a reduced risk) and unvaccinated (fully susceptible), by the beginning of the 2012–2013 epidemic (i.e., the time zero is the start of the epidemic and supplementary vaccination is assumed to be completed by that time). The population vaccinated is denoted as *S_v_*(*t*), and those who missed the chance to be immunized are denoted as *S_u_*(*t*). We assume that vaccine-induced immunity relatively reduces the susceptibility at a constant level, *ε* (vaccine efficacy). The numbers of people infectious and removed at time *t* are denoted, respectively, as *I*(*t*) and *R*(*t*). We then have differential equations describing the rate of change in each population as

(1) $$\begin{array}{l} \frac{dS_{u}}{dt}=-\lambda S_{u}I,\frac{dS_{v}}{dt}=-(1-\varepsilon )\lambda S_{v}I,\\ \frac{dI}{dt}=\lambda (S_{u}+(1-\varepsilon )S_{v})I-\gamma I,\\ \frac{dR}{dt}=\gamma I, \end{array}$$

where parameter *λ* is the transmission coefficient, and 1/*γ* is the mean infectious period. Given that the epidemic period is sufficiently shorter than the timescale of human demographic turnover, we did not consider background demographic dynamics, which led the population size preserved during the epidemic: $N=S_{u}\left(t\right)+S_{v}\left(t\right)+I\left(t\right)+R\left(t\right)$. The initial population are assumed to be categorized into either vaccinated susceptible or unvaccinated susceptible at the beginning of the epidemic: $S_{u}\left(0\right)+S_{v}\left(0\right)=N$ and *I*(0) = *R*(0) = 0.

Solving Equation (1), the differential equations with R(t) as an independent variable are integrated to

(2) $$S_{u}(t)=S_{u}(0)\exp\left[-\frac{\lambda }{\gamma }R(t)\right],\;S_{v}(t)=S_{v}(0)\exp\left[-\frac{\lambda }{\gamma }(1-\varepsilon )R(t)\right].$$

As the population size is preserved, we obtain the following relationship between the variables

(3) $$R(t)+S_{u}(0)\exp\left[-\frac{\lambda }{\gamma }R(t)\right]+S_{v}(0)\exp\left[-\frac{\lambda }{\gamma }(1-\varepsilon )R(t)\right]+I(t)=N.$$

Introducing the basic reproduction number, $R_{0}≔\lambda N/\gamma$, and the cumulative incidence, $z≔\underset{t\to +\infty }{\lim}R\left(t\right)/N$, we evaluate Equation (3) at the end of the epidemic (t→+∞) to have

(4) $$z+\frac{S_{u}(0)}{N}\exp\left[-R_{0}z\right]+\frac{S_{v}(0)}{N}\exp\left[-(1-\varepsilon )R_{0}z\right]=1,$$

which implies that once the initial susceptible population ($S_{u}\left(0\right),\;S_{v}\left(0\right)$), total population (N), and the basic reproduction number ($R_{0}$) are determined, the cumulative incidence (z) is determined as well. The cumulative incidence can then be used for evaluating the effectiveness and benefit-to-cost ratio. Equation (4) has a trivial solution *z* = 0 if

(5) $$\frac{S_{u}(0)}{N}\leq \frac{1-(1-\varepsilon )R_{0}}{\varepsilon R_{0}},$$

and otherwise, additional solution satisfying 0 < *z* < 1 is available.

### 2.2. Modelling Supplementary Vaccination Policies without Test Failure

We describe below the vaccination policies mathematically, considering the immunity status before supplementary vaccination. We specifically formulate the model for the case of test-and-vaccinate policy, due to its special feature of selective vaccination, but this modeling framework can also be applied to evaluating random mass vaccination by ignoring the selective feature of vaccination. To first simplify the example, we start mathematically implementing the supplementary vaccination, ignoring the failure of the laboratory testing (i.e., individuals with and without sufficient antibody are 100% detectable through the test).

**Figure 1:**
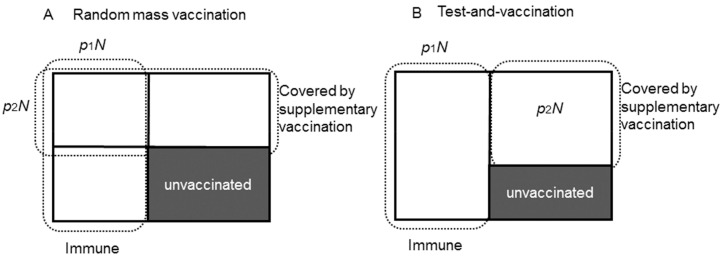
Different manners of reduction in unvaccinated individuals by supplementary vaccination. In advance of supplementary vaccination, it is assumed that *p*_1_*N* is already immune by routine vaccination (or natural infection), and supplementary vaccination is distributed to cover *p*_2_*N* of the population. The supplementary vaccination may be conducted either randomly (panel (**A**)) or following a negative outcome of serological testing (panel (**B**)). For simplicity, the serological testing is assumed to be perfect in this illustration. Panel B reduces unvaccinated individuals more effectively than panel (**A**).

Figure 1 shows the schematic illustration of the two different vaccination policies. Figure 1A corresponds to the random mass vaccination policy. $p_{1}$ is the proportion of immune population who had already acquired sufficient antibody from the routine vaccination program (or experienced past exposure to rubella) and $p_{2}$ is the proportion of the population who are covered by the supplementary vaccination. *N* is the total population. Assuming that people do not remember their own vaccination record of routine vaccination (which may not be realistic but considered as the worst-case scenario), routine and the supplementary vaccination are independent. In this case, some people ($p_{1}p_{2}N$) are covered by supplementary vaccination despite the fact that immunity was previously acquired. As a result, the population who remains unvaccinated after the implementation of this supplementary vaccination policy is ($1-p_{1})\left(1-p_{2}\right)N$. To avoid supplementary vaccination to be distributed to those who have previously acquired immunity by routine vaccination or past exposure to rubella, a test-and-vaccinate policy can be employed, where antibody test is performed before vaccination, and subjects without sufficient immunity are selectively vaccinated (Figure 1B). This policy would maximally enhance both individual-level immunity and herd immunity, in other words, $p_{1}N$ and $p_{2}N$ do not overlap, and the minimum unvaccinated population is realized: $\left(1-p_{1}-p_{2}\right)N$.

If the objective is simply to reduce the cumulative incidence at the end of the epidemic, test-and-vaccine strategy is the best, as it can minimize the unvaccinated population. However, if the objective is to reduce the economic burden of the epidemic (i.e., cost of treatment, opportunity cost lost due to infection, cost of vaccination, cost of antibody test), random vaccination could potentially be superior to selective strategy, due to the high cost of serological testing.

### 2.3. Modelling Vaccination Policies with Test Failure

In the section above, we ignored test failure, which is not realistic. Hereafter, we incorporate realistic performance of serological testing, which is described by two parameters, sensitivity (*α*) and specificity (*β*). 

**Figure 2:**
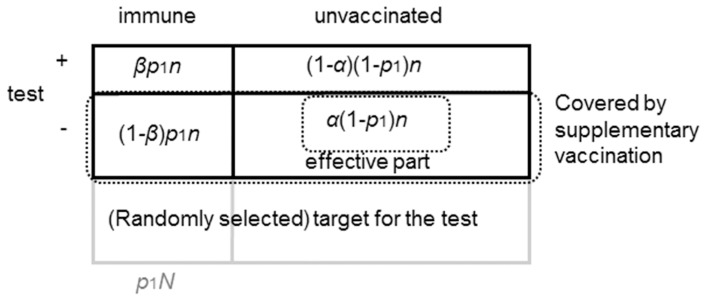
Performance of serological testing of immunity status against rubella. The target hosts are classified according to their immunity status before supplementary vaccination. The performance of serological testing is described by sensitivity *α* and specificity *β* with respect to the sero-negativity; i.e., sensitivity refers to the proportion that are test-negative among all the unvaccinated, and specificity is defined as the proportion that are test-positive among all vaccinated individuals. Accordingly, the number of people who have not been immune but vaccinated by the supplementary vaccination (true negatives) are given by *α*(1-*p*_1_)*n*.

Henceforward, it must be noted that we define sensitivity as the proportion tested negative among all unvaccinated individuals, and specificity is defined as the proportion tested positive among all vaccinated individuals. Figure 2 shows the classification of immunity status, and outcome of serological examination. *n* is the selected population for the serological test. Assuming random selection for the test, $p_{1}n$ are those already immune (from the routine vaccination or previous rubella infection), and $\left(1-p_{1}\right)n$ are those susceptible. The targets of supplementary vaccination are against those who tested seronegative, $\left(1-\beta \right)p_{1}n+\alpha \left(1-p_{1}\right)n$, but among the targeted population, only $\alpha \left(1-p_{1}\right)n$ are effectively contributing to an elevation of the herd immunity level.

As supplementary vaccination is conducted until the vaccine stock runs out, the number of supplementary doses consists of demand and supply 

(6) $$p_{2}N=\alpha (1-p_{1})n+(1-\beta )p_{1}n.$$

Solving this equation, *n* is represented by the other parameters 

(7) $$n=\frac{p_{2}N}{\alpha (1-p_{1})+(1-\beta )p_{1}}.$$

Given a realistic condition, *n* > *N*, the actual number of people who are newly vaccinated by supplementary vaccination are $\alpha \left(1-p_{1}\right)\min\left(n,N\right)$. Finally, the vaccinated and unvaccinated individuals before the epidemic (after the implementation of supplementary vaccination) are respectively represented as

(8) $$\begin{array}{l} S_{v}(0)=p_{1}N+\alpha (1-p_{1})\min(n,N),\\ S_{u}(0)=N-S_{v}(0). \end{array}.$$

Using this framework, other vaccination policies are realized by arranging the parameter values: no supplementary vaccination corresponds to *p_2_* = 0, and random mass vaccination scenario can be simulated when *α* = *β* = 1/2.

### 2.4. Cost-Benefit Analysis

Running the simulation of the above model, we obtain the cumulative incidence. Based on this, we compute the cost, C, and the benefit, B, of each vaccination policy. C is defined as the sum of cost from serological testing and vaccination, namely, $C_{vac}p_{2}N+C_{test}n$, where $C_{vac}$ and $C_{test}$ are the cost per a single test and a single dose of vaccination, respectively. B is defined as the reduced amount of the total disease cost given a case of rubella and CRS, namely, $m\left(z-z_{0}\right)NC_{CRS}+\left(z-z_{0}\right)NC_{rubella}$, where z and $z_{0}$ are the cumulative incidence with and without the implementation of the vaccination policy, and *C_rubella_* and *C_CRS_* are disease costs lost per case of rubella and CRS. Although we have not explicitly described CRS cases in the model, we assume that incidence of CRS is proportional to rubella incidence: $mz_{0}$ and mz with and without supplementary vaccination, respectively. As we focus on the benefit, we ignore adverse events that are known to be very rare.

### 2.5. Modelling Parameters and Simulation Settings

The above-described model is numerically simulated using assigned parameter values (Table 1).

First, we set the parameters which determines the epidemiological dynamics. The basic reproduction number ($R_{0}$) for rubella depends on the estimated country, and the estimation methods: 2.9–7.8 [13]. In this study, we set $R_{0}=6.0$. Vaccine efficacy was assumed as ε=0.90 [14]. Assuming serological testing exhibits a moderate performance, we adopt *α* = 0.85 and *β* = 0.80 for the performance of serological testing [15]. For supplementary vaccination coverage, value *p*_2_ = 0.2 was taken as a representative value [7]. Total population in Japan is assumed to be 120,000,000. The vaccinated fraction due to routine vaccination, *p*_1_, was varied from 0.0 to 1.0. The number of confirmed CRS cases during 2012–2013 season was 45 under supplementary vaccination policy [9]. Thus, we assume that the number of CRS cases per one rubella case is computed using cumulative incidence as follows: m=45/z.

Next, the following are the parameters used in the cost–benefit analysis. The lifetime disease cost of the CRS including treatments cost (direct cost) and loss of human capital due to CRS (indirect cost) was estimated as 139,910 USD in Oman, which is categorized as a high-income country. We used this cost as a proxy of that in Japan, converting it to Japanese Yen (JPY): 14,000,000 JPY (1 USD ≅ 100 JPY) [16]. A single measles-mumps-rubella (MMR) vaccine was estimated to cost 30 USD (≅3000 JPY) in the United States, and we set the vaccine cost at 1500 JPY per person, assuming price is discounted, since a substantial number of people is expected to undertake supplementary vaccination [16]. In Japan, serological testing currently costs 3000–6000 JPY, but the lower bound 3000 JPY was taken, given that substantial number of people undertake the same laboratory examination (Nishiura, personal communication). We have assigned 5000 JPY to *C_rubella_*, considering opportunity cost will be lost with rubella infection with the absence at school or workplace, and the cost was assumed to correspond to the average wage lost during the absence.

Based on above parameter settings, three different scenarios were considered for supplementary vaccination: (1) no implementation of vaccination, (2) random (non-selective) vaccination, and (3) test-and-vaccinate strategy, limiting vaccination to those who are seronegative (hereafter it is referred as “selective vaccination”). We used the statistical computing software R 3.3.1 for the simulation.

## 3. Results

### 3.1. Cumulative Incidence

**Figure 3:**
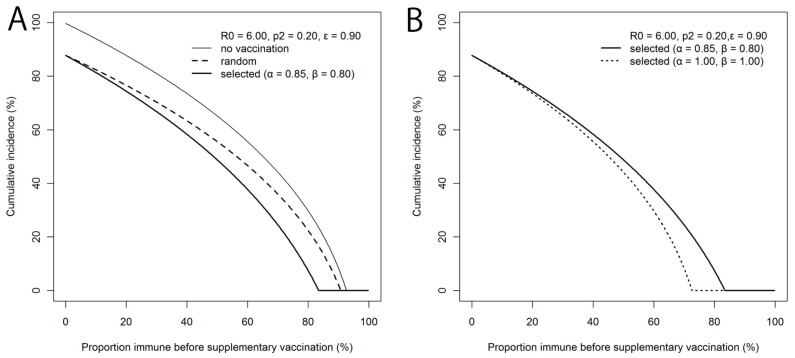
Cumulative incidence by different supplementary vaccination strategies. In panel (**A**), cumulative incidence is examined as a function of the fraction of the vaccinated in advance of supplementary vaccination, *p*_1_. When supplementary vaccination is implemented, it may be distributed randomly (thick dashed line), or selectively among sero-negative individuals (thick solid line). In panel (**B**), the similar cumulative incidence is examined in the event of selective supplementary vaccination under an assumption of perfect (dashed line) and imperfect serological testing (solid line). *R*_0_ is assumed to be 6.0. The supplementary vaccination coverage *p*_2_ is assumed to be 0.2. The efficacy of vaccination (ε) is assumed to be 90%.

Analytically solving the final size equation, Figure 3A shows the cumulative incidence *z* under the abovementioned setting, with three different scenarios of distributing supplementary vaccination as a function of the vaccination fraction *p*_1_. Critical values of vaccination *p_1_*_,crit_, which are determined by the condition of *z* to be larger than zero (*z* > 0 if *p*_1,crit_ < *p*) and calculated from Equations (5) and (8), are clearly identified in Figure 3, given different vaccination scenarios. The test-and-vaccinate strategy reduces the incidence most efficiently in comparison to other vaccination scenarios, even varying the proportion of vaccinated prior to the introduction of supplementary vaccination (*p*_1_). Moreover, the curve of the function for selective vaccination is even qualitatively well below other scenarios. In Figure 3B, the (*z*, *p*_1_)-curve for the selective vaccination under perfect serological testing (*α* = *β* = 1.0) is shown. This figure shows that *p*_1,crit_ = 72.5% should have been achieved if the antibody testing perfectly captured immunity status.

**Figure 4:**
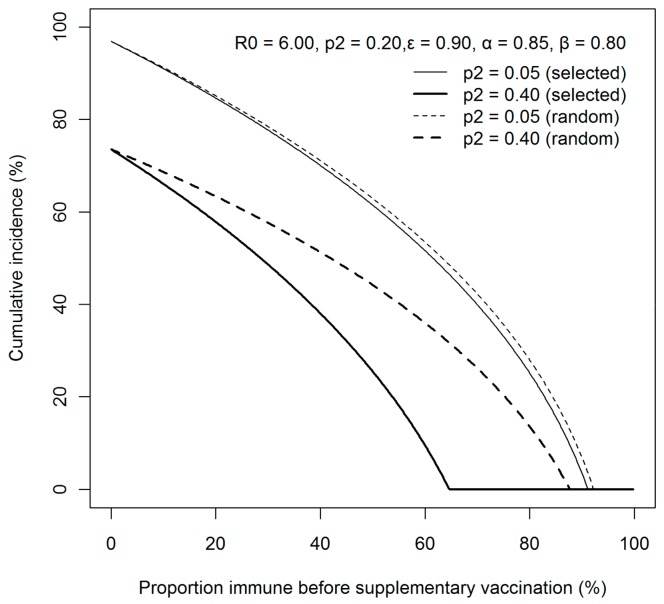
Sensitivity of the cumulative incidence of rubella to supplementary vaccination policy and coverage. Cumulative incidence is examined as a function of the fraction the vaccinated in advance of supplementary vaccination, *p*_1_. The supplementary vaccination is implemented either randomly (dashed lines) or selectively among sero-negative individuals (solid lines). *R*_0_ is assumed to be 6.0. The supplementary vaccination coverage *p*_2_ was either 5% or 40%. The efficacy of vaccination, *ε* was assumed to be 90%. Difference between supplementary vaccination strategies is notably visible for higher (0.40) supplementary vaccination coverage.

Figure 4 is the sensitivity analysis conducted, varying different possible supplementary vaccination coverages. The (*z*, *p*_1_)-curve using 0.05 and 0.40 as possible values for *p*_2_, are shown for both selective and random vaccination scenarios. The threshold level to prevent major epidemic greatly differs by vaccination strategy, and invariably, selective vaccination strategy is more efficient than random vaccination (e.g., *p*_1_,_crit_ = 64.7% at *p*_2_ = 0.40 applying selective vaccination, while the critical level is greater than 80% applying random vaccination).

### 3.2. Benefit-to-Cost Ratio Assessment

**Figure 5:**
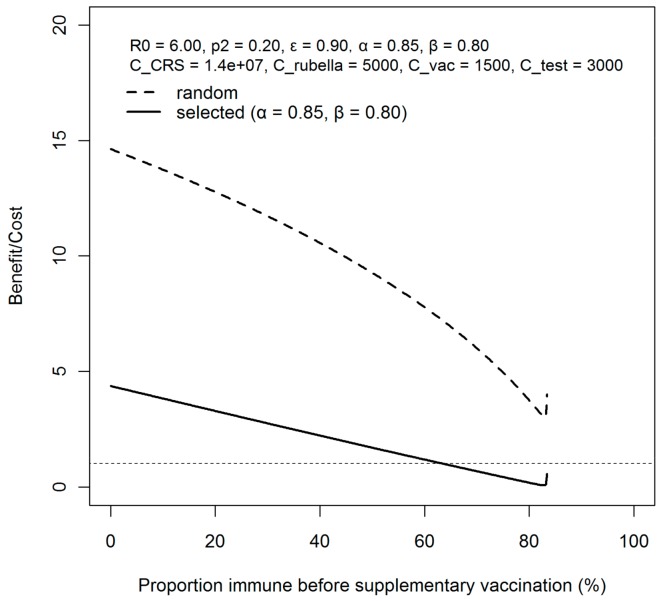
Comparison of cost-benefit ratios by supplementary vaccination strategies. The cost–benefit ratio is compared between random and selective supplementary vaccination strategies. *R*_0_ is assumed to be 6.0. The supplementary vaccination coverage *p*_2_ is set at 20%, while the efficacy of vaccination, *ε*, is assumed to be 90%. *C_CRS_* refers to the lifetime cost of congenital rubella syndrome, *C_rubella_* is the cost for rubella infection. *C_vac_* and *C_test_* are the cost of supplementary vaccination per person and serological testing per person. All the costs are expressed in Japanese Yen.

Figure 5 compares values of benefit to cost ratio (*B*/*C*) between the random and selective vaccination strategies, varying the proportion vaccinated before supplementary vaccination (*p*_1_). The value of (*B*/*C*] given random vaccination is much more effective than that of the selective vaccination for all *p*_1_ values. This has explicitly shown that random vaccination is not only relatively advantageous, but also cost efficient. The setting of *p*_1_ > *p*_1_,*_crit_* corresponds to the hypothetical situation that herd immunity had already been established in advance of the 2012–2013 epidemic.

**Figure 6:**
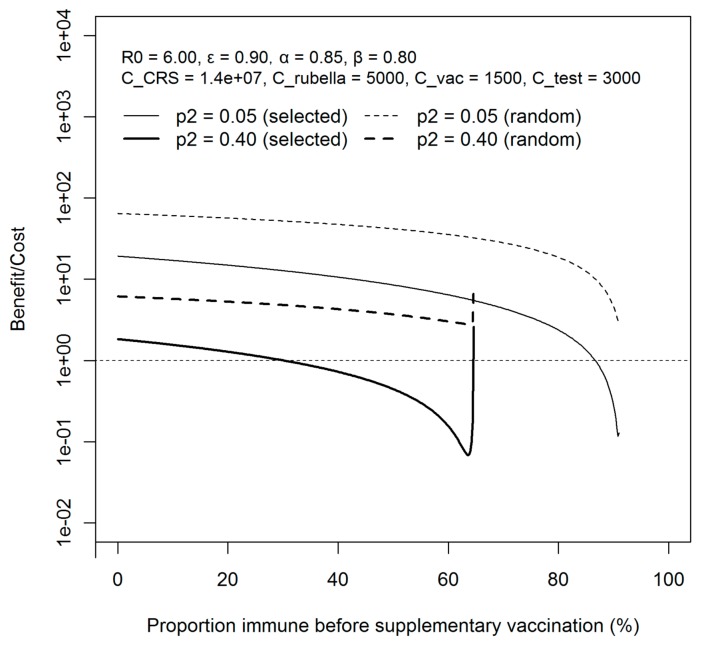
Comparison of the benefit-to-cost ratio by supplementary vaccination strategies. The cost–benefit ratio is compared between random and selective vaccination programs with the supplementary vaccination coverage of 5% and 40%. *R_0_* is assumed to be 6.0. The efficacy of vaccination, epsilon, is assumed to be 90%. *C_CRS_* refers to the lifetime cost of congenital rubella syndrome, *C_rubella_* is the cost for rubella infection. *C_vac_* and *C_test_* are the cost of supplementary vaccination per person and serological testing per person. All the costs are expressed in Japanese Yen. A logarithmic scale is chosen for easier comparison of (B/C) values.

To understand how sensitive the benefit-to-cost ratio would be to the fraction of people receiving supplementary vaccination, the coverage (*p_2_*) was varied (Figure 6). Similar (B/C) curves are observed as in Figure 5, applying 0.05 and 0.4 as an alternative value of *p_2_*. Still, the benefit-to-cost ratio when implementing random vaccination to the population is most beneficial, and the result does not drastically change under different parameter settings. Moreover, the value of (B/C) under random vaccination is consistently greater than selective vaccination when compared at the same supplemental vaccination fraction.

## 4. Discussion

Once a country decides to vaccinate the population against rubella, it is ideal that herd immunity is achieved to prevent the population from secondary transmission, and to also avoid the tragedy of newborn with CRS [17,18,19,20,21,22]. These published studies have shown that herd immunity is more beneficial than targeting women for individual protection purposes [17,18,19,20,21,22]. However, Japan has yet to achieve herd immunity, and thus, the transmission of the disease can continue in the present day [7]. Given supercritical susceptibility, the country experienced a large-scale epidemic from 2012 to 2013, and the Japanese government were advised to implement the test-and-vaccinate strategy due to the limited amount of RCV available in that year. Until herd immunity is achieved, Japan is continuously exposed to the risk of another major epidemic, with the possibility that the vaccine stockpile is insufficiently prepared. Using a mathematical model, the present study thus examined if the so-called test-and-vaccinate policy can be a realistic option in the future. We have shown that the effectiveness is greater applying selective supplementary vaccination than random vaccination. However, when we take the ratio of benefit to cost, the selective vaccination appeared to be too costly. Considering the imperfectness of diagnostic testing and the cost of the test itself in addition to the vaccines, the expenditure is not fully justifiable for the given benefit.

We have shown that substantial herd immunity is achievable by both vaccination strategies, but the condition is more efficiently satisfied by selective vaccination strategy. The differential efficiency was identified when the previously immune fraction is high and close to the critical level, which is in fact close to the situation in present Japan. Nevertheless, the cost of serological testing among healthy individuals is comparable with RCV, and is currently not covered by the National Health Insurance. Moreover, even when the immunity level was close to the critical level, applying selective vaccination strategy was more expensive than that of random vaccination. The proposed model can be used for any other settings, requiring revaccination following laboratory testing.

Despite experiencing the rubella epidemic in 2013, susceptible populations still remain in Japan, and supplementary vaccination is deemed essential for the country to fully appreciate the herd effect of the disease [23]. To achieve herd immunity with a restricted budget, this study has explicitly demonstrated that random vaccination is more beneficial. Thus, while identifying the characteristics of those susceptible is important, when planning future supplementary vaccination, strictly identifying the acquirement of immunity before vaccination is not economical.

Some limitations must be noted. First, the values of vaccines and serological test may change over time with introduction of new technologies. As the present study rests on mathematical models, we can demonstrate that qualitative results would not be varied by their variations within realistic parameter space (results not shown). Second, we did not consider the booster effect of revaccination for people with insufficient levels of immunity. Third, while a SIR model was applied, the dynamics of infection was not considered, except by the initial number of susceptibles and epidemic size. To fully capture the heterogeneous transmission dynamics, we did not consider age or gender in our model, which is crucial to estimate the burden of CRS, which we intend to address in another forthcoming study.

Nevertheless, this study gave a straightforward path for decision makers. The Japanese MHLW aims to eliminate rubella by 2020. While a high vaccination coverage is maintained among infants, adult males who missed the chance to acquire immunity remain able to sustain transmission. Random vaccination among adult males could be an economical option to achieve the goal by 2020.

## 5. Conclusions

We have assessed two strategies for the implementation of supplementary rubella vaccination, namely, random and selective vaccination, responding to the susceptibility status after the 2012–2013 epidemic in Japan [23]. The cumulative incidence and the benefit-to-cost ratio, which were employed as epidemiological and economic outcome measurements, allowed comparative assessments. In conclusion, the test-and-vaccinate policy reduces the number of cases efficiently, while effectiveness in terms of cost is lost, mainly since the cost of serological testing is inferior to random vaccination policy. Random vaccination is more efficient when a large stockpile of vaccination is available for distribution.

## Figures and Tables

**Table 1:** Parameter values.

**Parameters Determining Epidemiological Dynamics**				
**Description**	**Notation**	**Value**	**Unit**	**Reference**
Reproduction number	R_0_	6.0	non-dimensional	[13]
Total population in Japan	*N*	120,000,000	non-dimensional	Assumed
Vaccine efficacy	ε	0.90	non-dimensional	[14]
Sensitivity of the serological test	α	0.85	non-dimensional	[15]
Specificity of the serological test	β	0.80	non-dimensional	[15]
Proportion of the vaccinated before supplementary vaccination	*p*_1_	0.0–1.0	non-dimensional	Assumed
Proportion of supplementary vaccination coverage	*p*_2_	0.20	non-dimensional	Assumed
**Parameters Used in Cost-Benefit Analysis**				
**Description**	**Notation**	**Value**	**Unit**	**Reference**
Cost of serological test per person	*C*_test_	3000	JPY	Nishiura, personal communication
Cost of vaccination per person	*C*_vac_	1500	JPY	[16]
Cost for CRS	*C*_CRS_	14,000,000	JPY	[16]
Cost of rubella infection	*C*_rubella_	5000	JPY	Assumed

CRS: congenital rubella syndrome.
